# Fat-Free Mass Is Better Related to Serum Uric Acid Than Metabolic Homeostasis in Prader-Willi Syndrome

**DOI:** 10.3390/nu12092583

**Published:** 2020-08-25

**Authors:** Marzullo Paolo, Mele Chiara, Minocci Alessandro, Mai Stefania, Scacchi Massimo, Sartorio Alessandro, Aimaretti Gianluca, Grugni Graziano

**Affiliations:** 1 Division of General Medicine, IRCCS Istituto Auxologico Italiano, 28824 Piancavallo (VB), Italy; s.mai@auxologico.it (M.S.); massimo.scacchi@unimi.it (S.M.); 2 Department of Translational Medicine, Università del Piemonte Orientale, 28100 Novara, Italy; chiara.mele1989@gmail.com (M.C.); gianluca.aimaretti@med.uniupo.it (A.G.); 3 Division of Metabolic Diseases, IRCCS Istituto Auxologico Italiano, 28824 Piancavallo (VB), Italy; a.minocci@auxologico.it (M.A.); sartorio@auxologico.it (S.A.); 4 Division of Auxology, IRCCS Istituto Auxologico Italiano, 28824 Piancavallo (VB), Italy; g.grugni@auxologico.it

**Keywords:** Prader-Willi syndrome, uric acid, obesity, body composition, fat-free mass, resting energy expenditure, DXA

## Abstract

*Background*: Prader-Willi syndrome (PWS) is conventionally regarded as a model of genetic obesity carrying a metabolically healthier profile and fat compartmentalization than subjects with non-syndromic obesity. Serum uric acid (sUA) is a recognized surrogate marker of metabolic derangement. As no information is currently available on sUA levels in adults with PWS, we aimed to analyze sUA in a large cohort of adult patients with PWS in comparison to a control counterpart; secondly, we aimed to investigate the metabolic and non-metabolic determinants of sUA in PWS. *Methods*: A cross-sectional study was conducted on 89 consecutive adult patients with genetically confirmed PWS spanning a wide BMI range (17.2–56.7 kg/m^2^). As controls, 180 age-, sex- and BMI-matched healthy controls were included. sUA levels were analyzed in relation to the PWS status, metabolic variables, hormone status, body composition, and resting energy expenditure (REE). Bivariate correlation and multivariable regression studies were used to test for predictors of sUA in PWS. *Results*: Despite having similar BMI values, patients with PWS presented with higher FM (*p* < 0.0001), lower FFM (*p* < 0.0001) and REE values than controls (*p* < 0.0001). In PWS, sUA levels were non-significantly different between subjects with and without obesity (5.4 ± 1.3 vs. 4.9 ± 1.1 mg/dL, *p* = 0.09), and did not vary significantly in relation to genotype, sex steroid or GH replacement, as well as psychiatric treatments. Rates of hyperuricaemia (19.1% vs. 33.7%, *p* < 0.01) and absolute sUA levels were lower in patients with PWS compared to controls owing to significant differences between subgroups with obesity (5.5 ± 1.4 vs. 6.6 ± 1.6 mg/dL, *p* < 0.0001). In merged populations, sUA increased in parallel with age, BMI, FM, FFM, REE, glucolipid homeostasis, and inflammatory markers. In a separate analysis in PWS, however, sUA correlations with BMI, FM, and inflammatory markers were null. Stepwise multivariable regression analysis in the PWS group adjusted for karyotype, age, sex, FM, FFM, obesity, triglycerides, and HDL cholesterol, showed that sUA levels were independently associated with FFM (β = 0.35, *p* < 0.0001) and, albeit less significantly, with triglycerides (β = 0.23, *p* < 0.05). The introduction of height-normalized FFM (FFM index) in the regression model, however, abrogated the predictive role of FFM on sUA. *Conclusions*: FFM mass is a strong predictor of sUA. PWS is associated to lower sUA levels than controls likely due to genetic predisposition to different body composition and healthier metabolic phenotype. Further studies are warranted to assess purine metabolism and the clinical significance of the FFM index in PWS.

## 1. Introduction

Prader-Willi syndrome (PWS) originates from a defective paternal chromosome 15q11.2–q13 and comprises three main genotypes: paternal 15q11–q13 deletion (type I or II, depending on the proximal breakpoint); maternal uniparental disomy (UPD15); and imprinting defects [1]. With an overall estimated prevalence of 1:10,000–1:30,000, the complex phenotype of PWS embraces a number of neurodevelopmental defects, evolving from muscle hypotonia and failure to thrive in the neonatal stage to progressive developmental impairment, learning disabilities and dysmorphic features during early growth [2]. Often, patients with PWS manifest behavioral problems and obsessive craving for food, which predisposes many patients to severe obesity if hyperphagia is left unrestrained [3]. Delayed motor development and decreased motor performance with impaired muscle mass persist through childhood into adulthood in relation to abnormal body composition and neuromuscular functioning [4]. Also frequent in PWS are complex hypothalamic and endocrine disorders, which predominantly affect the somatotropic and gonadotropic axis for their lifespan [5,6].

In adults, PWS shows an atypical body composition featured by adiposity with subcutaneous fat accumulation, neuromuscular alterations with muscle hypotrophy and hypotonia, which further contribute to scarce propensity to exercise, poor physical performance, and susceptibility to adiposity [4,7,8,9,10]. Despite a trend toward metabolic impairment, the metabolic burden of PWS subjects is somewhat milder than that expected in relation to obesity degree, in terms of insulin resistance [11,12,13], chronic inflammation [14], liver steatosis [15], and metabolic syndrome [16]. Biological mechanisms propelling this apparently advantageous metabolic setting involve peripheral rather than central adiposity [7], diverse metabolomic signatures of adipocytes [17], and distinct profiles of insulin-sensitizing hormones [11,18,19,20,21].

Serum uric acid (sUA) has been long investigated for its dual role as a bystander and promoter of metabolic derangement [22]. Metabolic impairment and obesity are recognized allies of hyperuricemia along with dietary consumption of purine-rich food and insulin resistance, with this latter contributing to reducing sUA clearance [23,24]. Moreover, adipose tissue and, particularly, visceral fat expressed xanthine oxidoreductase, the enzyme catalyzing purines to UA [25]. To date, no study has systematically analyzed sUA and its regulation with respect to genetic, anthropometric, and clinical features of PWS. A previous report in a small cohort of obese patients with PWS allowed us to show lower sUA than BMI-matched controls [20]. The present investigation sought to study sUA levels in a large group of adults with PWS spanning a wide BMI range, to test the potential influence of genetic, anthropometric, and biochemical factors on sUA.

## 2. Materials and Methods

### 2.1. Patients

For this study, 89 consecutive patients with PWS were prospectively enrolled. Descriptive data are summarized in the results section Table 1. A diagnosis of PWS was performed on typical clinical features and molecular genetic studies of chromosome 15. We documented 15q11–q13 deletion in 67 (75.2%) and UPD15 in 21 patients (23.6%), while 1 patient had a positive methylation test. As controls, 180 age-, sex, and BMI matched subjects were recruited among the hospital staff and patients admitted to our institution for work-up and rehabilitation of obesity. Following an initial assessment, patients with PWS and controls were included in a 1:2 ratio in an effort to compensate for the syndromic presentation of PWS in terms of body composition and clinical features after excluding subjects with kidney or liver disease, autoimmune disorders, uncontrolled hypothyroidism, pre-existing diabetes mellitus, exposure to glucocorticoids, neoplasms and hematological diseases. For all participants, daily alcohol consumption was estimated at <125 mL. Other exclusion criteria included medications directly interfering with UA levels and hypertriglyceridemia. It should be reminded, however, that only 19 patients with PWS were free of pharmacological treatments; in the remainder, endocrine treatments included sex steroids in 33, growth hormone in 22, levothyroxine in 11, and cortisone in 2 cases; other therapies encompassed triclyclic antidepressants and antipsychotic therapies in 28, SSRI in 9, anti-hypertensive drugs in 11, bisphosphonates in 2, thionamides in 1 and DOAC in another case. Among controls, all women were premenopausal as assessed by a personal history of regular menses, and 21 were taking oral estro-progestins. All subjects, or their parents/guardians for patients with PWS, gave their informed consent for inclusion before they participated in the study. The study was conducted in accordance with the Declaration of Helsinki, and the protocol was approved by the Ethics Committee of Istituto Auxologico Italiano (ref. no. 01C025; acronym: PWSIPMET).

### 2.2. Body Measurements

All subjects underwent body measurements wearing light underwear, in fasting conditions after voiding. Weight and height were measured to the nearest 0.1 kg and 0.1 cm, respectively, using standard methods. BMI was expressed as body mass (kg)/height (m^2^). Obesity was defined for any BMI over 30 kg/m^2^. Waist circumference (WC) was determined in standing position midway between the lowest rib and the top of the iliac crest after gentle expiration, with a non-elastic flexible tape measure.

The respiratory quotient (RQ; VO_2_/VCO_2_) and resting energy expenditure (REE; kcal/24 h) were determined in a thermo-regulated room (22–24 °C) by computed open-circuit indirect calorimetry, measuring resting oxygen uptake and resting carbon dioxide production by a ventilated canopy (Sensormedics, Milan, Italy) at 1-min intervals for 30 min and expressed as a 24-h value. All tests were performed between 07:30 and 09:00 a.m. after voiding, with patients in fasting condition, after avoiding any physical and non-physical activity (smoking, drinking) potentially interfering with an appropriate calorimetric assessment. The test was conducted by certified nurses and consists of making each patient lie down relaxed on a comfortable armchair, with the head under a transparent hood connected to a pump, which applies adjustable ventilation through it. Exhaled gas dilutes with the fresh air ventilated under the hood and a sample of this mixture is conveyed to the analyzers, through a capillary tube and analyzed. Ambient and diluted fractions of O_2_ and CO_2_ are measured for a known ventilation rate, and O_2_ consumption (VO_2_) and CO_2_ production (VCO_2_) are determined. Energy expenditure (EE) was calculated according to Weir’s equation:

EE = 5.68 VO_2_ + 1.59 VCO_2_ − 2.17 N_u_ (where N_u_ is urinary nitrogen).

As short-term urinary collections to assess total nitrogen excretion (N_u_) may not be representative of the protein oxidized during the measurement itself, they were not obtained in this study and assumed to be 13 g/24 h [26]. The predicted REE (pREE) was calculated by the Harris-Benedict formula and allowed to test for metabolic efficiency, calculated as the ratio between measured/predicted REE values, as previously reported [27].

A dual-energy X-ray absorptiometry (DEXA; GE Lunar, Madison, WI, USA) was performed for the assessment of body composition. Exams were performed by certified radiological technologists blinded with regards to the study protocol and laboratory data. Daily calibrations of the machine were conducted as per manufacturer’s instructions. Total and regional scans were taken for each participant. For this study, percent body fat mass (% FM) and fat-free mass (FFM, kg) were considered of interest.

### 2.3. Laboratory

Blood samples were drawn under fasting conditions. Blood levels of UA, glucose, total cholesterol, high density (HDL), and low-density (LDL) lipoprotein cholesterol and triglycerides were measured by enzymatic methods (Roche Diagnostics, Mannheim, Germany). Serum insulin levels were measured using a Cobas Integra 800 Autoanalyzer (Roche Diagnostics, Indianapolis, IN, USA). Ultrasensitive C-reactive protein was measured by CRP (latex) HS Roche kit. Hyperuricemia was defined as above 7 mg/dL in men and above 5.7 mg/dL in women. Insulin resistance was calculated by the homeostasis model of assessment-insulin resistance (HOMA-IR) approach, calculated as insulin (microunits per milliliter) x blood glucose (millimoles per liter)/22.5 [28].

### 2.4. Statistical Analysis

Statistical analysis was performed using SPSS version 21 (Somers, NY, USA). Values are expressed as mean ± SE or percentage. Data points not normally distributed obtained by the Shapiro-Wilk test were log-transformed to improve the symmetry and homoscedasticity of the distribution. For comparative analysis, the Student’s *t*-test for unpaired data and analysis of variance for parametric and Mann–Whitney test for nonparametric data were used as appropriate.

Pearson’s correlation analysis and the chi-square test were used to identify significant associations between variables of interest. Using merged and separate datasets from study groups, binary logistic and stepwise multivariable regression analyses were developed to test the relationship of hyperuricemia, PWS status, or sUA levels with a number of independent variables, i.e., PWS karyotype (DEL15 = 0; UPD15 = 1), age, sex (female = 0, male = 1), BMI or obesity (BMI > 30 kg/m^2^), percent FM and FFM expressed in kilograms. Regression models were developed to avoid collinearity. Odds ratio, β coefficients and related significance values obtained from the models are reported. Significance was set at *p* < 0.05.

## 3. Results

### 3.1. Anthropometric Results

Mean BMI values ranged between 17.2–56.7 kg/m^2^ in patients with PWS and 18.7–59.4 kg/m^2^ in controls. Among patients with PWS, 16.8% were normal-weight (BMI, 22.8 ± 1.9 kg/m^2^), 14.6% were overweight (BMI, 27.1 ± 1.5 kg/m^2^), and 68.5% were obese (BMI, 39.9 ± 6.6 kg/m^2^), with the overall rate of severe obesity (BMI > 40 kg/m^2^) being 29.2%. As shown in Table 1, patients with PWS presented with higher FM but lower FFM and REE values than controls (*p* < 0.0001 for all). Compared to controls, subjects with PWS had a higher proportion of FM both in cases without (PWS, 37.0 ± 6.2%; Controls, 25.2 ± 6.9%; *p* < 0.0001) and with obesity (PWS, 47.9 ± 5.4%; Controls, 44.2 ± 6.3%; *p* < 0.001). Nonetheless, patients with PWS showed lower levels in total and LDL-cholesterol than controls.

### 3.2. Analysis of Serum Uric Acid

**Figure 1:**
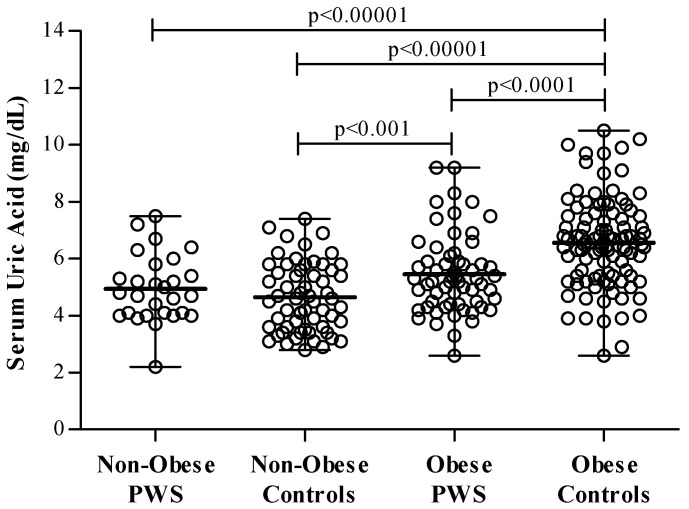
Scatter dot plot (mean and ranges) of serum uric acid levels in PWS and controls grouped as non-obese and obese (BMI ≥ 30 kg/m^2^).

No PWS or control subject reported previous or current symptoms of hyperuricemia. In PWS, sUA levels ranged between 2.2–9.2 mg/dL and were, on average, lower than in controls, particularly when stratified by the presence of obesity (Figure 1). Mean sUA levels were similar between non-obese PWS and controls (4.9 ± 1.2 vs. 4.7 ± 1.2 mg/dL), while they were significantly lower in PWS patients with obesity compared to controls with obesity (5.5 ± 1.4 vs. 6.6 ± 1.6 mg/dL, *p* < 0.0001).

Hyperuricemia was diagnosed in 28% of cases, e.g., 19.1% of PWS and 33.7% of control subjects (χ^2^ = 10.2, *p* < 0.01). In PWS, there were borderline differences in sUA levels (5.4 ± 1.3 vs. 4.9 ± 1.1 mg/dL, *p* = 0.09) and hyperuricemia (24.5% vs. 7.1%; χ^2^ = 3.8, *p* = 0.052) between patients with and without obesity, while differences in sUA according to the presence of obesity were wider in controls (sUA: 6.6 ± 1.2 vs. 4.5 ± 1.5 mg/dL, *p* < 0.00001; 49% vs. 10.2%; χ^2^ = 21.4, *p* = 0.0001). In analysis restricted to the PWS group, males exhibited higher sUA than females (5.7 ± 1.4 vs. 4.8 ± 1.1 mg/dL, *p* = 0.003) but hyperuricemia rates were similar between genders (16.2% vs. 21.7%; *p* = 0.5). 

In a subanalysis on 12 PWS subjects (13.4%) with metabolic syndrome defined according to the Treatment Panel III report [29], sUA levels were higher than those without (6.3 ± 1.8 vs. 5.1 ± 1.1 mg/dL, *p* < 0.01). Analysis by genotype did not reveal differences in sUA between DEL15 and UPD15 patients (5.2 ± 1.2 vs. 5.3 ± 1.8 mg/dL). Patients with PWS who were on GH therapy at the time of the study exhibited slightly higher sUA levels compared to GH non-users (5.7 ± 1.4 vs. 5.1 ± 1.2 mg/dL, *p* = 0.08) likely due to differences in FFM (47.2 ± 7.5 vs. 37.8 ± 6.8 kg, *p* < 0.00001). Alternatively, no influence of sex steroid and psychiatric treatments on sUA was seen (data not shown).

### 3.3. Correlation Analyses

Expected associations related sUA to indices of adiposity, glucolipid homeostasis, and inflammation, both in merged and separated populations (Table 2).

**Figure 2:**
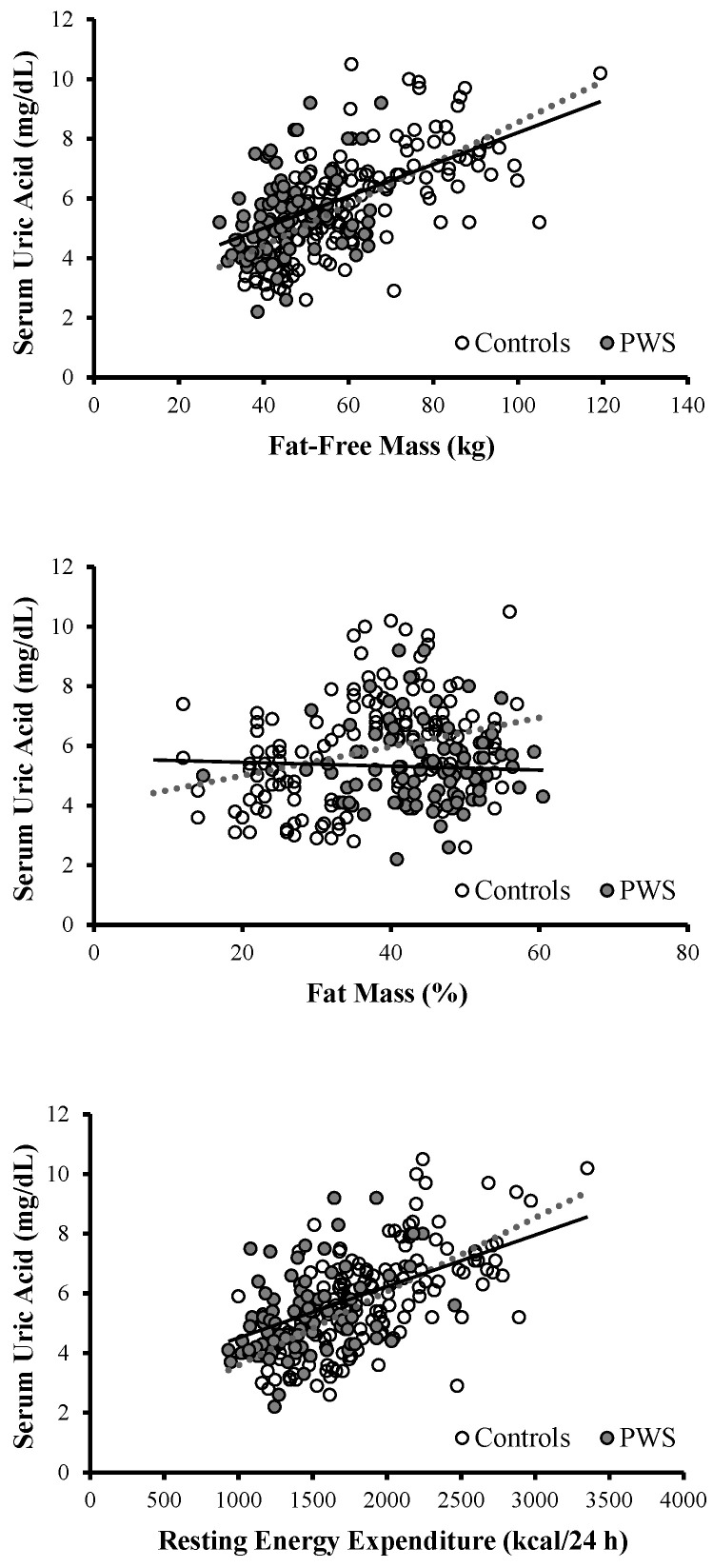
Bivariate correlation analysis between serum UA levels and resting energy expenditure (upper panel), fat-free mass (middle panel), and fat mass (lower panel). Closed circles and closed line: patients with PWS; open circles and dashed line: control subjects.

However, sUA was more tightly associated with FFM than metabolic variables or FM (Figure 2), and each increase in one kg in FFM was associated with a 4.4% higher likelihood of hyperuricemia (odds ratio = 1.044; 95%CI, 1.004–1.086; *p* < 0.05). 

A direct association was also seen between sUA and REE (Figure 2), but adding FFM as a covariate weakened this relationship (whole population: *r* = 0.14, *p* < 0.05; PWS: *r* = 0.19, *p* = 0.09; controls: *r* = 0.17; *p* < 0.05).

A multivariable regression model was subsequently used to test predictors of sUA. Based on the results of bivariate regression analysis, a number of equations were computed to build the most predictive regression model, particularly around the use of FFM or FFMI. FFM and triglyceride levels emerged as significant associates of sUA levels (Table 3). 

Interestingly, when FFMI was used instead of FFM, different variables entered the regression equation, namely gender (β = 0.26, *p* < 0.01), obesity (β = 0.30, *p* < 0.01), age (β = −0.25, *p* < 0.05) and tryglicerides (β = 0.20, *p* < 0.05). It is also worth noting that when REE was added to the regression equation, it became the only predictor of sUA (β = 0.35, *p* < 0.01), but this association disappeared when REE was normalized by FFM. In the control group, variables entering the regression equation included obesity (β = 0.41, *p* < 0.0001), gender (β = 0.27, *p* < 0.01), and FFM (β = 0.26, *p* < 0.05).

## 4. Discussion

The results of our study show that sUA levels are lower in PWS adults and, overall, less influenced by obesity and metabolic derangement than in a control population of BMI-matched subjects. In PWS, fat-free mass and triglycerides emerged as significant predictors of sUA, suggesting a role for sUA as a measure of muscle purine degradation in this genetic setting. Surprisingly, when the height-normalized fat-free mass was accounted for, fat-free mass no longer acted as a predictor of sUA in PWS.

sUA level represents a metabolic gauge acknowledged for its ability to predict cardiovascular risk in relation to adiposity, unbalanced diet, and metabolic derangement [30]. In the general US population, the rate of asymptomatic hyperuricemia is 21% [31], yet the progression to clinically overt gout affects 20% of these cases in the presence of risk factors, such as an unhealthy diet, obesity and exaggerated alcohol consumption [32]. Our study aimed to describe sUA levels and its determinants in PWS, a genetic condition predisposing to neurodevelopmental defects, severe obesity, and premature mortality [33]. To overcome analytical caveats relating to this rare disease, e.g., small representative samples, diverging developmental stages, obesity degrees, and ongoing replacement therapies, we enrolled a discrete sample of patients who were tested in comparison to a significantly larger control sample, in the effort to compensate for known syndromic peculiarities affecting PWS. Clinically speaking, no patients with PWS and/or their families reported prior, present, or recurring symptoms of hyperuricemia, nor we detected a direct effect of genotype, gonadal replacement, and psychiatric treatments in determining hyperuricemia. A trend toward an association between GH therapy and hyperuricemia occurred, but this was clinically meaningless and likely driven by higher muscle mass achieved in GH users. Although >80% of our PWS subjects were overweight or obese, sUA levels were lower than BMI-matched controls, and this difference achieved significance between obese subgroups. Hyperuricemia affected 20% of PWS subjects. While this proportion exceeds the 12% rate reported for the general Italian population [34], it remains significantly lower than the 34% rate detected in the control group. Expectedly, sUA was associated with metabolic health and, in PWS, triglycerides emerged as a metabolic predictor of sUA at the multivariable regression analysis. Literature pertaining to sUA regulation in PWS is poor and restricted to case reports [35,36] and a preliminary study in obese adults with PWS, where sUA level was lower than control subjects with obesity [20]. In the general population, metabolic health and, particularly, triglycerides are recognized to play a modulatory role on sUA in relation to the cardiovascular risk [37,38,39]. Evidence exists that sUA levels are better correlated with visceral adiposity, insulin resistance, and liver steatosis than with subcutaneous fat accumulation [40,41,42]. Moreover, xanthine oxidoreductase, the enzyme that catalyzes purines to UA, is overexpressed in adipose tissue in parallel with visceral accumulation of fat [25]. In our PWS cohort, however, the waist was not an associate of sUA. This result supports previous findings showing that the waist is not a reliable metabolic predictor in PWS due to peripheral fat partitioning with a predominant subcutaneous accumulation of abdominal adipose tissue [12]. Together, our findings prompt the suggestion that metabolic health is a determinant of sUA in PWS but highlights the potential importance of differences in fat distribution between PWS and controls as a determinant of metabolic health [43].

In seeking for physiological determinants of sUA, an examination of our dataset outlined a robust correlation between sUA and fat-free mass. In PWS, a fat-free mass was the strongest correlate of sUA, while fat mass and inflammatory markers showed no association, a notion that is clinically relevant for PWS in relation to metabolic derangement, inflammation and gout [44]. In the general population, the direction of the association between sUA and fat-free mass is debated. Previous cross-sectional studies reported either positive associations between increasing sUA and lean mass in pubertal children [45] as well as middle-aged and elderly adults [46,47], or positive associations between high sUA and an increasing degree of sarcopenia [48,49]. Skeletal muscle, the largest body source of purine [50], is impaired in PWS due to innate and acquired quantitative and qualitative abnormalities. As confirmed herein, fat-free mass is decreased in PWS and this is known to be associated with neuromuscular alterations encompassing muscle hypotrophy and hypotonia [1,2,3,4,6,8,9,10]. Studies on muscle ultrastructure revealed an impairment of type-2 and type-2B fast-twitch fibers in PWS, along with an increase in immature type-2C fibers [4,51,52]. Given that that type-2 fibers harbor the largest ATP content and largely contribute to sUA production [53,54], it could be hypothesized that muscle hypotrophy and type-2 fiber be linked to impaired nucleotide degradation, hence sUA production, in comparison to non-PWS controls. Although the introduction of a height-adjusted fat-free mass index, which virtually compensates for differences in height between persons [55], abrogated the predictive role of fat-free mass on sUA, it should be emphasized that this index does not seem to perform metabolically better than other anthropometric measures in PWS [56]. Moreover, its clinical value is potentially disadvantaged by several disorders affecting these patients with PWS, such as (1) abnormalities in muscle development, tone, strength, structure, ultrastructure; (2) intrinsic defects of neuro-sensitive and neuro-motor functions; (3) the multifactorial origin of short stature; (4) interindividual inhomogeneity for past/current exposure to GH [1,2,3,4,6,8,9,10,51,52]. As population-specific formulae are required to estimate fat-free mass (FFM) in obese subjects with PWS [57], we envisage that the clinical significance of the fat-free mass index in PWS be further assessed in specifically designed future studies. With regards to the tight association between sUA and resting energy expenditure, this was mutually related to fat-free mass and likely reflected the large use of ATP as the energy source for contraction. It has long been shown that resting energy expenditure varies among people independent of body size and composition and that skeletal muscle metabolism is the main determinant of metabolic rate [58]. Because de novo purine biosynthesis is a high-energy event [25], sUA could provide an indirect estimate of ATP degradation to signal ATP availability, hence the energy state, to other organs or apparatuses [59], like it has been recently hypothesized for non-PWS individuals [60]. Further studies are warranted to verify if muscle abnormalities in PWS can affect metabolic activities involving ATP production vs. hydrolysis.

This investigation has important limitations. UA excretion was not measured in urine, therefore we could not assess the effect of UA retention in obese vs. lean individuals. Also, we did not measure ATP levels and urinary purine excretion, hence we cannot clarify the potential role of sUA as a measure of ATP degradation and purine metabolism. Moreover, the lack of dietary intake assessment cannot exclude different consumption of purine-rich food in PWS as a potential explanation of our results. Fourthly, the cross-sectionality design of the study does not allow us to assess the cause-effect relationship. And even after enrolling a much larger control group to compensate for the peculiar body composition of PWS adults, an exact matching with control subjects was impossible to realize. Despite these weaknesses that are partly explained by its retrospective nature, this is the first study assessing sUA in a fairly large population of adults with genetically confirmed PWS and spanning a wide range of BMIs, using an accurate analysis of body composition and basal metabolism, and counting multiple anthropometric and biochemical parameters. In conclusion, sUA is a surrogate of metabolic health in PWS and reflects with fair accuracy the fat-free mass and energy state of our patients. Further studies are needed to clarify if this link is functional or adaptive in response to abnormal substrates oxidation in PWS.

## 5. Conclusions

While PWS predisposes to genetic obesity and serum UA is seen as a conventional predictor of cardiometabolic derangement, hyperuricemia is less prevalent in an adult population with PWS with a high prevalence of obesity, when compared to a BMI-matched control population. Fat-free mass is a strong determinant of serum uric acid levels in PWS, potentially due to peculiarities in muscle structure and muscle mass.

## Figures and Tables

**Table 1:** Summary of anthropometric, metabolic, and biochemical parameters in patients with Prader-Willi syndrome (PWS) and control subjects. Significance was calculated by Mann-Whitney and chi-square tests.

Parameters	PWS Group (*n* = 89)	Control Group (*n* = 180)	*p*
Sex (M/F, %)	48/52	50/50	0.6
Age (year)	28.4 ± 8.7	29.8 ± 7.5	0.2
Weight (kg)	83.7 ± 20.7	100.5 ± 32.2	0.0001
Height (cm)	154.8 ± 9.2	169.4 ± 9.8	0.0001
BMI	35.1 ± 9.0	34.2 ± 9.4	0.4
Waist (cm)	107.5 ± 17.7	104.2 ± 24.4	0.2
WHR	0.92 ± 0.08	0.87 ± 0.11	0.01
FM (%)	44.5 ± 7.5	37.3 ± 11.1	0.0001
FFM (kg)	45.3 ± 9	60.8 ± 16.2	0.0001
FFMI	16.7 ± 2.9	20.9 ± 4.1	0.0001
REE (kcal/24 h)	1455 ± 310	1895 ± 433	0.0001
REE/pREE (%)	85.0 ± 10.1	99.4 ± 10.2	0.0001
REE_FFM_ (kcal/24 h/kg)	36.5 ± 5.1	31.9 ± 3.7	0.0001
RQ	0.87 ± 0.07	0.87 ± 0.10	0.9
Blood glucose (mg/dL)	86.0 ± 8.9	88.5 ± 25.3	0.3
Insulin (mIU/L)	10.9 ± 7.6	12.2 ± 7.2	0.2
HOMA-IR	2.3 ± 1.7	2.6 ± 2.0	0.1
Uric Acid (mg/dL)	5.2 ± 1.3	5.8 ± 1.7	0.008
Cholesterol (mg/dL)	183.7 ± 34.0	199.9 ± 38.9	0.0001
HDL (mg/dL)	51.8 ± 14.5	52.3 ± 20.0	0.8
LDL (mg/dL)	121.0 ± 33.1	131.1 ± 34.2	0.03
Triglycerides (mg/dL)	120.9 ± 34.2	143.3 ± 94.0	0.0001
CRP (mg/dL)	0.58 ± 0.83	0.76 ± 0.83	0.053
Fibrinogen (mg/dL)	358.8 ± 82.1	367.3 ± 81.5	0.8

For abbreviations: BMI, body mass index; WHR, waist-to-hip ratio; FM, fat mass; FFM, fat-free mass; FFMI, FFM index calculated as FFM [kg]/height squared [m^2^]; REE, resting energy expenditure; REE/pREE, REE/predicted REE ratio; REE_FFM_, REE normalized by FFM; RQ, respiratory quotient; HOMA-IR, a homeostatic model of insulin resistance; UA, uric acid; CHO, total cholesterol; HDL, high-density lipoprotein; LDL, low-density lipoprotein; CRP, C-reactive protein.

**Table 2:** Pearson’s correlation analysis between sUA levels (mg/dL) and variables of interest in merged and separated study groups. The correlation coefficient r and significance are presented. For significance: ^a^, *p* < 0.05; ^b^, *p* < 0.01; ^c^, *p* < 0.001; ^d^, *p* < 0.0001.

Parameters	Whole Population	PWS Group	Control Group
Age (years)	−0.15 ^a^	−0.17	−0.17
BMI (kg/m^2^)	0.42 ^d^	0.11	0.57 ^d^
Waist (cm)	0.63 ^d^	0.18	0.80 ^d^
WHR	0.38 ^d^	0.22 ^a^	0.51 ^d^
FM (%)	0.16 ^a^	−0.08	0.31 ^d^
FFM (kg)	0.59 ^d^	0.39 ^d^	0.65 ^d^
FFMI	0.56 ^d^	0.25 ^a^	0.65 ^d^
REE (kcal/24 h)	0.63 ^d^	0.37 ^d^	0.63 ^d^
REE/pREE	0.05	0.16	−0.24 ^b^
REE_FFM_	−0.18 ^b^	0.01	−0.23 ^b^
RQ (VO_2_/VCO_2_)	−0.15 ^a^	−0.17	−0.15
Glucose (mg/dL)	0.05	0.28 ^b^	0.01
Insulin (mIU/mL)	0.37 ^d^	0.27 ^b^	0.42 ^d^
HOMA-IR	0.33 ^d^	0.30 ^b^	0.34 ^d^
CHO (mg/dL)	0.07	0.08	0.03
HDL (mg/dL)	−0.39 ^d^	−0.29 ^b^	−0.42 ^d^
LDL (mg/dL)	0.17 ^c^	0.17	0.14
Triglycerides (mg/dL)	0.33 ^d^	0.29 ^b^	0.33 ^d^
CRP (mg/dL)	0.18 ^b^	0.16	0.26 ^c^
Fibrinogen (mg/dL)	0.15 ^a^	0.07	0.18 ^a^

For abbreviations: BMI, body mass index; WHR, waist-to-hip ratio; FM, fat mass; FFM, fat-free mass; FFMI, FFM index calculated as FFM [kg]/height squared [m^2^]; REE, resting energy expenditure; REE/pREE, REE-to-predicted REE ratio; RQ, respiratory quotient; HOMA-IR, homeostatic model of insulin resistance; CHO, total cholesterol; HDL, high-density lipoprotein; LDL, low-density lipoprotein; CRP, C-reactive protein.

**Table 3:** Stepwise multivariable regression analysis in the PWS group. sUA was tested as the dependent (continuous) variable. Independent variables tested in the equation were age (year), sex (females = 0, males = 1), obesity (BMI > 30 kg/m^2^), PWS karyotype (DEL15 = 0, UPD15 = 1), fat-free mass (FFM, kg), triglycerides (mg/dL), and HDL-cholesterol (mg/dL) levels.

Dependent Variable	Independent Variables	Beta	T	*p*	Adjusted *R*^2^
sUA (mg/dL)	FFM	0.35	3.66	0.0001	0.19
	Triglycerides	0.23	2.44	0.017	
	Age	0.18	−1.93	0.056	
	Karyotype	0.10	1.06	0.3	
	HDL	0.11	−1.05	0.3	
	Sex	0.08	0.72	0.4	
	Obesity	0.02	0.22	0.8	
